# Supplementary material for: Fabrication and Characterization of Hydrophobic Cellulose Nanofibrils/Silica Nanocomposites with Hexadecyltrimethoxysilane
Source: Polymers (Basel). 2022 Feb 21;14(4):833. doi: 10.3390/polym14040833 (PMC8963035; doi:10.3390/polym14040833)
Supplement: Supplementary file 1 [file polymers-14-00833-s001.zip › polymers-1586324-supplementary.pdf]

# Fabrication and Characterization of Hydrophobic Cellulose Nanofibrils/Silica Nanocomposites with Hexadecyltrimethoxysilane

**Gi-Hong Kim [1], Dong-Ho Kang [1], Bich-Nam Jung [1,2] and Jin-Kie Shim [1,*]**

[1] Korea Packaging Center, Korea Institute of Industrial Technology, Bucheon 14449, Korea; kakamate@kitech.re.kr (G.-H.K.); kangppp@kitech.re.kr (D.-H.K.); jbn5666@kitech.re.kr (B.-N.J.)
[2] Department of Chemical and Biological Engineering, Korea University, Seoul 02841, Korea
* Correspondence: jkshim@kitech.re.kr; Tel.: +82-32-650-4771

## 1. Morphology of the CNF

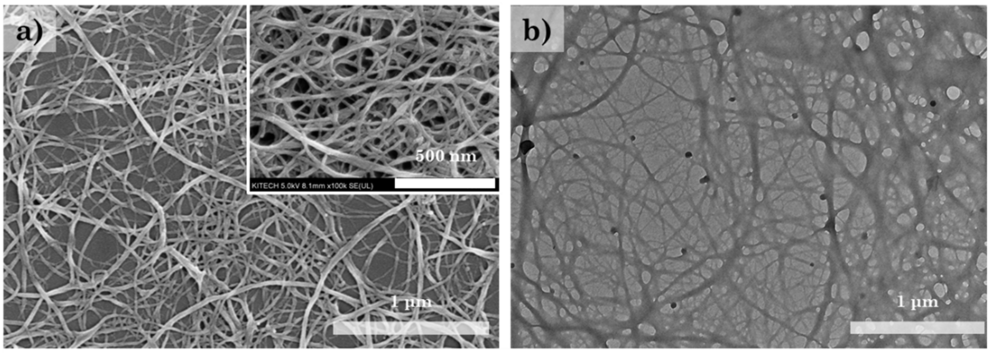


**Figure S1**. Morphology of the CNF investigated via (**a**) SEM and (**b**) TEM.

## 2. Calculation of the CNF, CNF/Silica, and Poly-HDTMS Content of the CNF Composites

Table S1 shows the elemental composition of the CNF surface before and after modification with TEOS and HDTMS obtained via XPS. The CNF, silica, and HDTMS content was calculated using the following equations based on each XPS elemental analysis. Here, the Si coefficient ($k$) of HDTMS was assumed to be 1.

$$CNF = (C_6H_{10}O_5)_n = x,\ 162.14\ g/mol$$

$$Silica = SiO_2 = y,\ 60.08\ g/mol$$

$$HDMTS = CH_3(CH_2)_{15}SiO_k = z,\ 269.58\ g/mol\ (Assuming\ k = 1)$$

$$C = 6x+16z$$

$$O = 5x + 2y + kz$$

$$Si = y + z$$

$$z = \frac{(6O - 5C - 12\,Si)}{(6k - 92)}$$

$$x = \frac{(C - 16z)}{6}$$

$$y = Si - z$$

**Table S1**. Surface composition of the samples measured via XPS.

| Samples | Atomic percentage (%) | | | CNF(wt%) | Silica(wt%) | HDTMS (wt%) |
|---|---|---|---|---|---|---|
| | C | O | Si | | | |
| CNF | 56.20 | 43.80 | - | 100.00 | - | - |
| CNF/silica | 29.20 | 54.65 | 16.15 | 44.85 | 55.15 | - |
| h-sCNF 0.1 | 33.94 | 50.97 | 15.05 | 40.65 | 51.18 | 8.17 |
| h-sCNF 0.5 | 52.82 | 35.78 | 11.40 | 30.14 | 34.04 | 35.82 |
| h-sCNF 1.0 | 57.14 | 31.85 | 11.01 | 24.98 | 31.09 | 43.92 |
| h-sCNF 2.0 | 64.82 | 25.66 | 9.52 | 20.29 | 23.53 | 56.18 |
| h-CNF 0.4 | 67.30 | 30.55 | 2.15 | 60.53 | - | 39.46 |
| h-CNF 0.8 | 66.71 | 31.37 | 1.92 | 65.27 | - | 34.73 |

## 3. Calculation of the CNF, CNF/silica, and Inorganic/Organic HDTMS Content of the CNF Nanocomposites

The inorganic/organic HDTMS amount was calculated using the CNF, silica, and HDTMS amount based on the XPS analysis shown in Table S2 and the thermal degradation properties ($T_{max}$) obtained from the TGA. Here, the inorganic/organic HDTMS content of h-CNF and h-sCNF samples was calculated according to the ash content of the CNF and CNF/silica nanocomposites. Firstly, the $T_{max}$ value of CNF/silica was 57.41 wt%, which is about 2.26 wt% higher compared with the silica content of the CNF due to the insulating effect of the silica nanoparticles. Therefore, the ach content of the CNF/silica nanocomposites was assumed to be 2.26 wt%.

$$Ash\ of CNF/silica, a = T_{max,CNF/silica} - Silica\ content_{CNF/silica,XPS}$$

$$=57.41 - 55.15 = 2.26\ wt\%$$

$$Ash\ of\ CNF,\ b = T_{max,CNF} = 0.88\ wt\%$$

$$Inorganic\ HDTMS_{h-sCNF} = T_{max,h-sCNF} - Silica\ content_{h-sCNF,XPS} - 2.26$$

$$Organic\ HDTMS_{h-sCNF} = HDTMS_{h-sCNF,XPS} - Inorganic\ HDTMS_{h-sCNF}$$

$$Inorganic\ HDTMS_{h-CNF} = HDTMS_{h-CNF,XPS} - T_{max,h-CNF} - 0.88$$

$$Organic\ HDTMS_{h-CNF} = HDTMS_{h-CNF,XPS} - Inorganic\ HDTMS_{h-CNF}$$

**Table S2.** Thermal degradation properties ($T_{max}$) and amount of the CNF, silica, and inorganic/organic HDTMS.

| Samples | TGA | XPS | | Inorganic HDTMS (wt%) | Organic HDTMS (wt%) |
|---|---|---|---|---|---|
| | $T_{max}$ (wt%) | Silica (wt%) | HDTMS (wt%) | | |
| CNF | 0.88 | - | - | - | - |
| CNF/silica | 57.41 | 55.15 | - | - | - |
| h-sCNF 0.1 | 55.02 | 51.18 | 8.17 | 1.58 | 6.59 |
| h-sCNF 0.5 | 50.77 | 34.04 | 35.82 | 14.47 | 21.35 |
| h-sCNF 1.0 | 46.54 | 31.09 | 43.92 | 13.19 | 30.73 |
| h-sCNF 2.0 | 38.03 | 23.53 | 56.18 | 12.24 | 43.94 |
| h-CNF 0.4 | 14.34 | - | 39.46 | 13.46 | 26.00 |
| h-CNF 0.8 | 17.93 | - | 34.73 | 17.05 | 17.68 |

## 4. Thermal Properties of the CNF/Silica and HDTMS-Modified CNF/Silica Nanocomposite

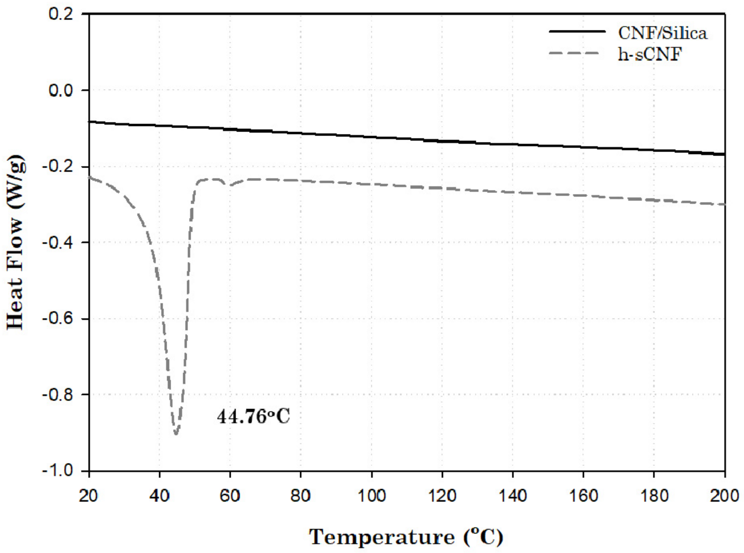


**Figure S2**. DSC curves of the CNF/silica and HDTMS-modified CNF/silica nanocomposites.
